# Non-invasive prenatal diagnosis (NIPD): how analysis of cell-free DNA in maternal plasma has changed prenatal diagnosis for monogenic disorders

**DOI:** 10.1042/CS20210380

**Published:** 2022-11-16

**Authors:** Britt Hanson, Elizabeth Scotchman, Lyn S. Chitty, Natalie J. Chandler

**Affiliations:** 1 North Thames Genomic Laboratory Hub, Great Ormond Street NHS Foundation Trust, London, U.K.; 2 Genetic and Genomic Medicine, UCL Great Ormond Street Institute of Child Health, London, U.K.

**Keywords:** Cell-free fetal DNA, cffDNA, Monogenic disorder, NIPD, Non-invasive, Prenatal

## Abstract

Cell-free fetal DNA (cffDNA) is released into the maternal circulation from trophoblastic cells during pregnancy, is detectable from 4 weeks and is representative of the entire fetal genome. The presence of this cffDNA in the maternal bloodstream has enabled clinical implementation of non-invasive prenatal diagnosis (NIPD) for monogenic disorders. Detection of paternally inherited and *de novo* mutations is relatively straightforward, and several methods have been developed for clinical use, including quantitative polymerase chain reaction (qPCR), and PCR followed by restriction enzyme digest (PCR-RED) or next-generation sequencing (NGS). A greater challenge has been in the detection of maternally inherited variants owing to the high background of maternal cell-free DNA (cfDNA). Molecular counting techniques have been developed to measure subtle changes in allele frequency. For instance, relative haplotype dosage analysis (RHDO), which uses single nucleotide polymorphisms (SNPs) for phasing of high- and low-risk alleles, is clinically available for several monogenic disorders. A major drawback is that RHDO requires samples from both parents and an affected or unaffected proband, therefore alternative methods, such as proband-free RHDO and relative mutation dosage (RMD), are being investigated. cffDNA was thought to exist only as short fragments (<500 bp); however, long-read sequencing technologies have recently revealed a range of sizes up to ∼23 kb. cffDNA also carries a specific placental epigenetic mark, and so fragmentomics and epigenetics are of interest for targeted enrichment of cffDNA. Cell-based NIPD approaches are also currently under investigation as a means to obtain a pure source of intact fetal genomic DNA.

## Introduction

Rare diseases are a heterogenous group of disorders each typically affecting less than one in 2000 people within the general population [1]. More than 6000 unique rare diseases have been entered in the Orphanet database to date, the majority of which arise from inherited or *de novo* single-gene anomalies that result in chronic and sometimes life-threatening implications for patients [1]. The three main classes of rare monogenic disorders are those which have autosomal recessive (AR), autosomal dominant (AD), and X-linked modes of inheritance, with an estimated prevalence of 35.02%, 23.78% and 5.63%, respectively, and thus accounting for more than 60% of all rare genetic diseases [1]. While many of these disorders are not currently curable or preventable, several therapies and interventions have been developed which, if implemented *in utero* or at an early postnatal timepoint, could improve the quality of life of affected individuals. As such, prenatal testing has played a pivotal role in shaping pregnancy management as well as the clinical outcome of patients with rare monogenic disorders, either by allowing early initiation of therapy or by allowing parents choices with regard to pregnancy management. Historically, access to the fetal genotype has relied upon invasive sampling methods (i.e. amniocentesis and chorionic villus sampling [CVS]) which have been thought to carry a small but significant risk of miscarriage (∼0.1–0.3%) [2]. Results from a recent meta-analysis, however, suggest that there is no increased risk associated with amniocentesis or CVS [3], but outcomes depend on the skill of the clinician offering testing and this can be variable. Even so, findings from extensive patient surveys suggest that less invasive procedures are preferred where available [4–6], necessitating continued focus towards development of methods that enable non-invasive prenatal diagnosis (NIPD) of single-gene disorders.

**Figure 1:**
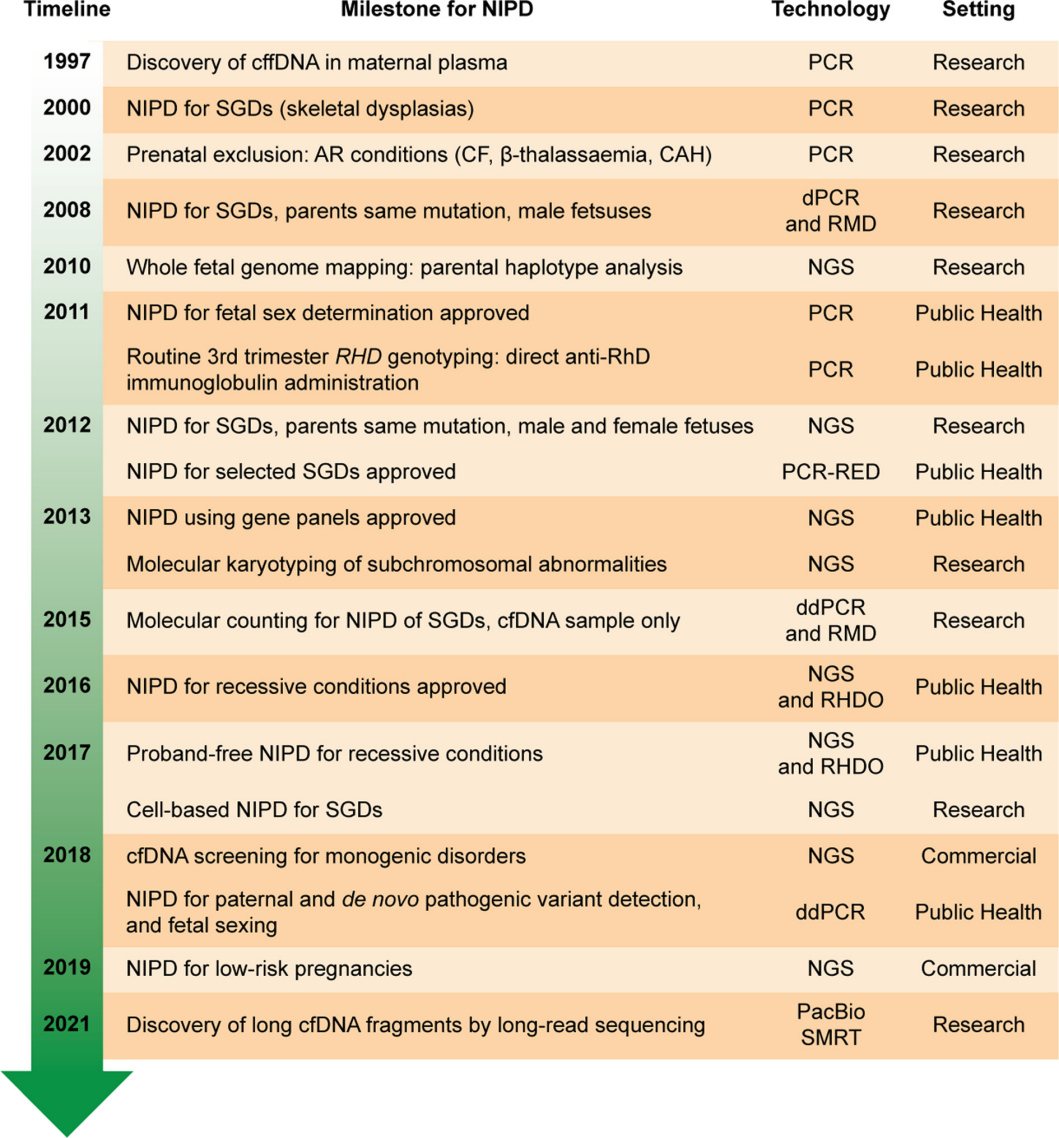
Timeline of NIPD milestones Timeline showing the major milestones reached for NIPD in the research, clinical, and commercial settings since the discovery of cffDNA in maternal plasma. Abbreviations: AR, autosomal recessive; cfDNA, cell-free DNA; ddPCR, droplet digital PCR; dPCR, digital PCR; NGS, next-generation sequencing; PacBio SMRT, Pacific Biosciences single molecule real-time sequencing; PCR, polymerase chain reaction; PCR-RED, PCR followed by restriction enzyme digest; * RHD*, rhesus D blood group status; RMD, relative mutation dosage; RHDO, relative haplotype dosage analysis; SGD, single-gene disorder.

In 1997, analysis of cell free DNA (cfDNA) identified Y-chromosome genomic material circulating in the blood of pregnant women carrying male fetuses [7] (Figure 1). We now know that cell-free fetal DNA (cffDNA) is representative of the entire fetal genome [8] and, as such, has provided unprecedented access to the fetal genome through non-invasive sampling (i.e. venous blood draw). cffDNA originates from placental trophoblastic cells [9], is shed into the maternal bloodstream in a highly fragmented form and is detectable from as early as 4 weeks of gestation [10,11]. The average length of cffDNA fragments has been reported to be ∼143 bp, and maternal cfDNA ∼166 bp, as a result of fragmentation within the linker regions flanking nucleosomes, or at the nucleosome binding sites, respectively [8,12]. Crucially, this value has recently been suggested to be biased by the use of short-read sequencing technologies, and longer cfDNA fragments (ranging from 500 bp to ∼23 kb) have since also been detected in maternal circulation as a result of developments in long-read sequencing technologies [13] (Figure 1). Yu and colleagues reported that the proportion of fragments over 500 bp were approximately 15%, 20% and 32% across the three trimesters of pregnancy, respectively [13].

In addition to the non-invasive aspect of cffDNA acquisition for prenatal diagnosis and screening, a further advantage of this method is the stage of pregnancy at which samples can be obtained. Invasive methods are typically carried out from 11 weeks of gestation onwards [14], whereas reliable levels of cffDNA for non-invasive clinical testing can theoretically be obtained from ∼7 weeks, as is the case for fetal sexing [15], providing additional valuable time for diagnosis and informing choices for pregnancy management [16,17]. The quantity of cffDNA in circulation is otherwise referred to as the fetal fraction (FF), and the fetal DNA is cleared from maternal circulation shortly after birth making it specific to ongoing pregnancy [18]. In a study measuring cfDNA obtained from 1949 high- and low-risk singleton pregnancies, the average FF at 11–13 weeks of gestation was reported to be ∼10%, increasing by 2.6% per week from 8 to 10 weeks, 0.2% per week between 10 and 20 weeks, and 0.7% per week thereafter [19]. The authors reported that FF was positively correlated with gestational age and negatively correlated with maternal weight [19], although there is still limited consensus on the precise aetiology of FF variability across individual pregnancies [20]. In addition to gestational age, maternal weight/body mass index (BMI) [21] and natural biological variation [19], several other contributing factors have been suggested, including fetal aneuploidy (thought largely to be related to placental size), and pregnancy complications (and related pathology).

A decade following the discovery of cffDNA, Down syndrome was detected by analysis of cfDNA in the maternal circulation in a pregnancy where the fetus had Trisomy 21 (T21) [22]. Since then, non-invasive prenatal testing (NIPT) via cfDNA has been implemented clinically in more than 60 countries worldwide [23], specifically to screen for the common aneuploidies (i.e. T13, T18 and T21) [24]. NIPT for aneuploidy requires confirmation of a positive result by invasive testing because of the risk of discordant results which may be caused by confined placental mosaicism (CPM), the existence of maternal chromosomal rearrangements, maternal neoplasia-derived cfDNA, and cffDNA released from a vanishing twin [25,26]. Conversely, NIPD for single gene diseases in families at known increased risk does not require follow-up testing as maternal genomic DNA is accounted for during the analysis, and CPM is extremely rare in monogenic disorders [27].

**Figure 2:**
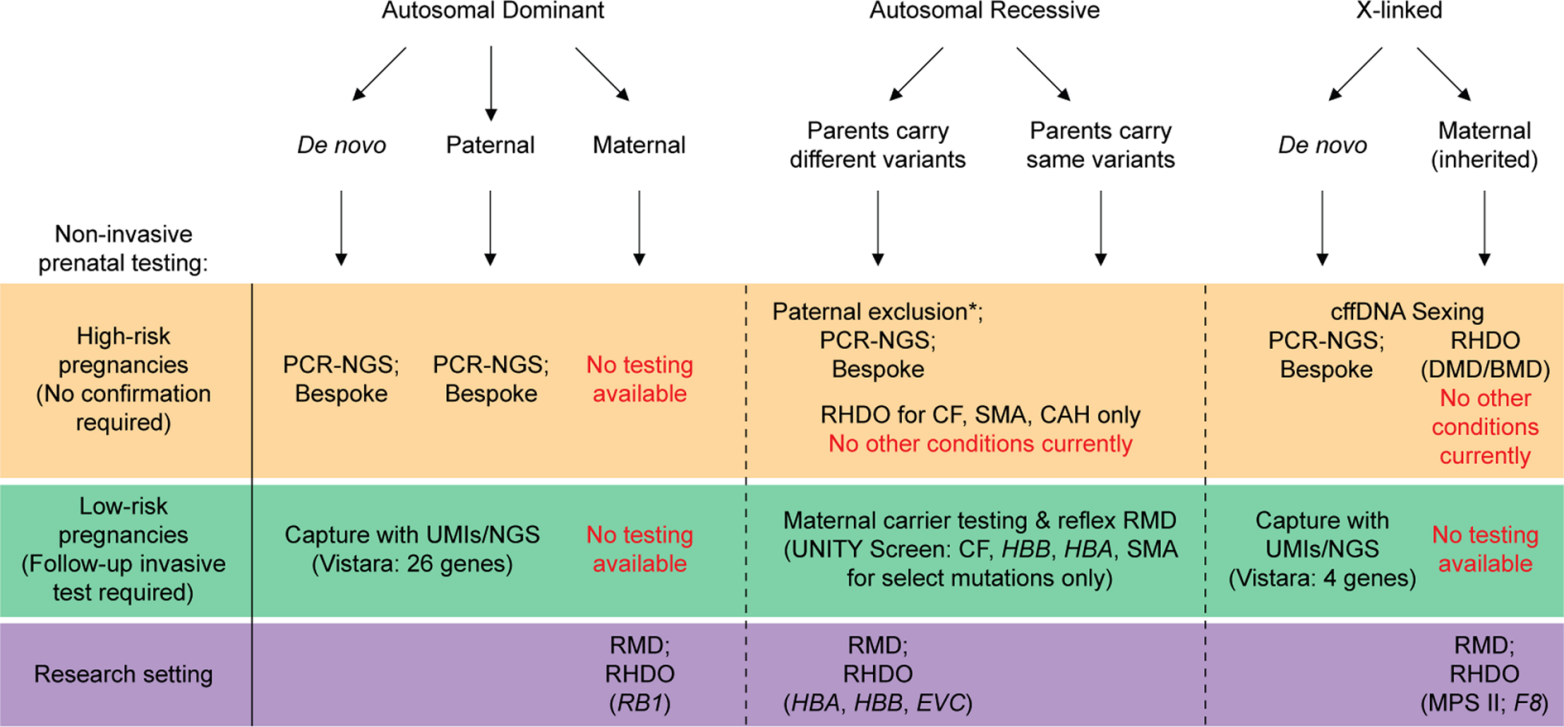
Non-invasive prenatal tests available and in development for diagnosis of monogenic disease Diagram depicting the different tests that are currently available either in the public health or commercial settings for high- and low-risk pregnancies, as well as those which are in development in the research setting. Invasive follow-up testing is required for low-risk pregnancy screening using the commercially available tests (i.e. Vistara and UNITY Screen), or for paternal exclusion in instances where the paternal pathogenic variant is detected in high-risk pregnancies (*). Abbreviations: CAH, congenital adrenal hyperplasia; CF, cystic fibrosis; cffDNA, cell-free fetal DNA; DMD/BMD, Duchenne/Becker muscular dystrophy; *EVC*, gene mutations causing Ellis van Creveld syndrome;* F8*, gene mutations causing factor 8 disorder/haemophilia A;* HBA* and *HBB*, gene mutations causing α- and β-thalassaemia respectively; MPS II, Mucopolysaccharidosis type II/Hunter syndrome; PCR-NGS, polymerase chain reaction followed by next-generation sequencing; *RB1*, gene mutations causing retinoblastoma; RHDO, relative haplotype dosage analysis; RMD, relative mutation dosage; SMA, spinal muscular atrophy; UMI, unique molecular identifier.

The first research papers describing NIPD for monogenic disorders, namely achondroplasia [28] and myotonic dystrophy [29], were published in 2000 (Figure 1). Over the years, a number of authors have published reports of cfDNA analysis for a variety of monogenic disorders, but use in clinical service has largely been confined to fetal sex determination [30,31] and rhesus D blood group (*RHD*) status [32,33] in *RHD* negative women (Figure 1). Since, the repertoire of monogenic disorders that can be tested for using NIPD has increased to include *FGFR2-* and *FGFR3*-related syndromes [34–37], cystic fibrosis (CF) [38,39], spinal muscular atrophy (SMA), Duchenne and Becker muscular dystrophies (DMD, BMD) [40–42], as well as bespoke testing for families with rare mutations [43] (Figures 1 and 2). Although the aggregated incidence of monogenic disorders is relatively high, individually cases are rare, and this has hindered the widespread clinical implementation of bespoke testing. Additionally, the high background of maternal cfDNA is a significant challenge with regards to working with cffDNA, particularly for the detection of maternally inherited variants.

Expanded NIPD for monogenic disorders is not only important for informing the reproductive choices of prospective parents but also for health service planning and provisioning. This review will cover the tests that are currently in clinical service for NIPD of monogenic disorders in high-risk pregnancies, as well as the strategies that are being developed towards future clinical implementation, and includes a brief overview of the tests which are commercially available for NIPT in low-risk pregnancies.

## NIPD technologies

### Paternally inherited and *de novo* variants

#### qPCR and PCR-RED

A relatively straightforward approach to NIPD is for the detection of paternally inherited or *de novo* variants which are, by definition, not present in the maternal fraction of cfDNA [6]. Several PCR-based methods have been used, including real-time quantitative PCR (RT-qPCR) for fetal sex determination. Digital PCR (dPCR) has also recently been implemented clinically for the purpose of identifying male fetuses in families at risk for X-linked disorders where conventional qPCR approaches are not applicable [44], and for paternal mutation exclusion testing whereby the absence of the paternal variant indicates that the fetus is not affected [45] (Figure 1). Determination of fetal sex using non-invasive methods reduces unnecessary further testing or treatment, such as in the instance of a female pregnancy in families at risk of X-linked disorders (e.g. BMD/DMD [30]) (Figure 2), and in cases of congenital adrenal hyperplasia (CAH) where treatment with dexamethasone can be restricted to female fetuses [46,47]. Some of the first tests for NIPD of monogenic disease to be delivered clinically were based on PCR followed by restriction enzyme digestion (PCR-RED), specifically for the diagnosis of two *FGFR3* disorders (achondroplasia and thanatophoric dysplasia) [34,35]. There are several limitations to the PCR-RED approach, however, including that it is gene- and variant-specific, requires gel-based analysis which can be subjective, is not applicable to all mutations or for cases where the causative gene is unknown, and it has an inconclusive rate of 8% [35].

#### PCR and NGS

Targeted sequencing of specific genomic loci through PCR followed by next-generation sequencing (PCR-NGS) has emerged as a more sensitive and accurate NIPD approach to conventional PCR-based methods, with a broader mutation detection range for single gene disorders (Figures 1 and 2). This approach can be used to screen for all variants within a gene, for example, autosomal dominant *FGFR2*- and *FGFR3*-related conditions [37]. Recently, PCR-NGS has been used for the prediction of fetal ABO blood group [48]. For autosomal recessive disorders, such as CF [38] and β-thalassaemia [49], PCR-NGS is employed for paternal mutation exclusion (Figures 1 and 2) whereafter detection of the paternal allele requires invasive follow-up testing to determine inheritance of the maternal variant. Additionally, accredited clinical services using PCR-NGS are available for bespoke testing of paternally inherited mutations in families with a known risk for a particular monogenic disease, or where a *de novo* mutation has been detected in a previous pregnancy [27,37,38] (Figures 1 and 2). This approach, however, has not yet been widely clinically implemented owing to its costly and labour-intensive nature.

### Autosomal recessive and inherited X-linked disorders

While detection of *de novo* or paternally inherited variants in cfDNA is relatively straightforward, accurately assigning maternally inherited variants poses a significant challenge owing to the high background of maternal plasma DNA. PCR-NGS is not fully quantitative owing to relatively low read depths, and thus is unable to assign maternally-inherited variants to the FF. This instead requires the use of highly sensitive techniques that can accurately detect small imbalances between wild-type (WT) and pathogenic variants, such as relative haplotype dosage analysis (RHDO) and relative mutation dosage (RMD) (Figures 1 and 2). Newer molecular counting techniques are also being developed to facilitate NGS-based maternal variant detection in cfDNA, discussed in more detail below.

#### RHDO

**Figure 3:**
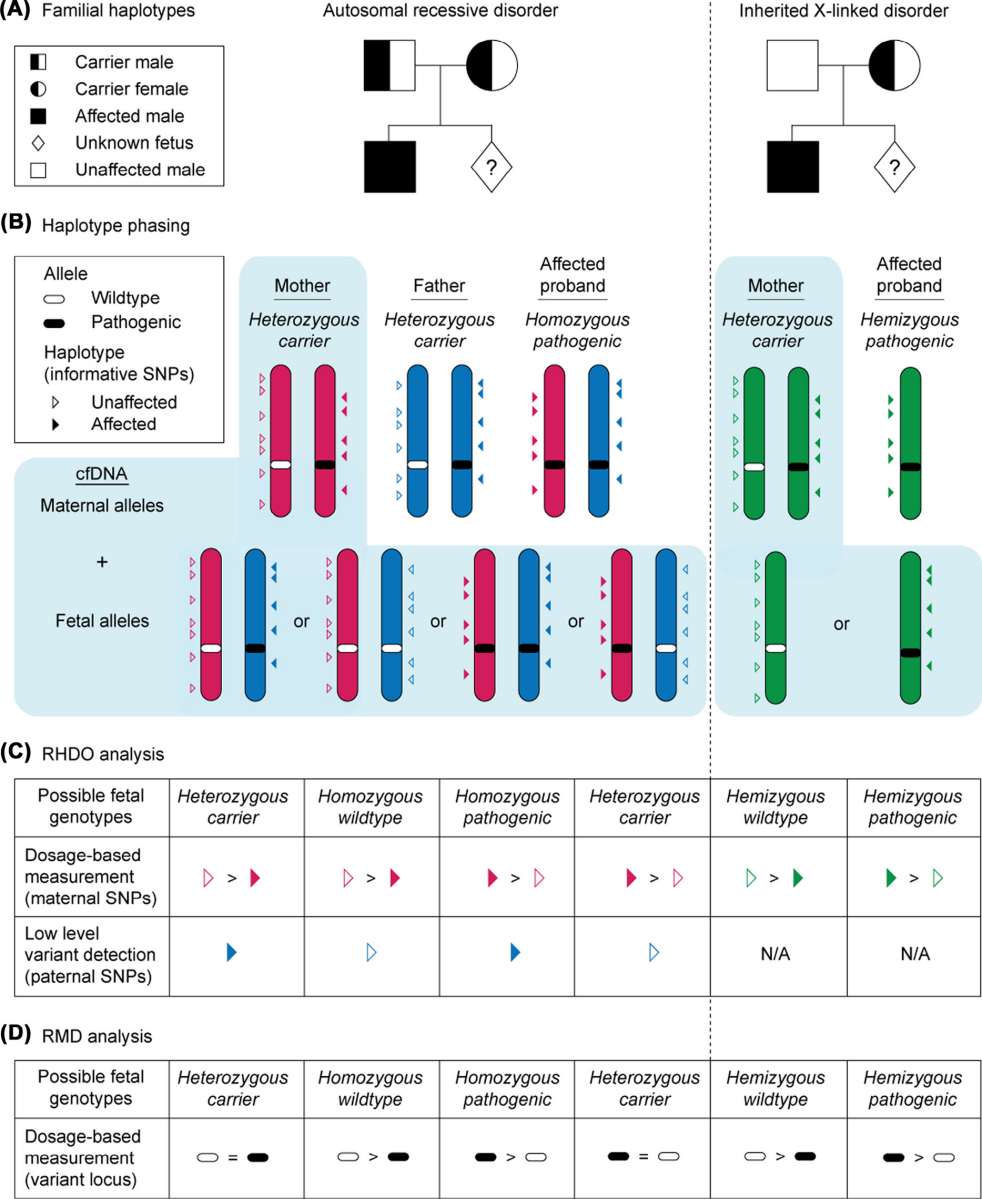
Schematic overview of RHDO and RMD for NIPD of AR and inherited X-linked conditions (**A**) Representative family trees depicting possible familial haplotypes for AR and inherited X-linked conditions. Left hand panel: family tree shows parents that are heterozygous carriers for an AR condition, with an affected child (i.e. the proband) of which the sex is not relevant, and an unborn fetus of unknown genotype. Right hand panel: an unaffected father and a mother who is a carrier for an X-linked disorder, with an affected male child (i.e. the proband) and an unborn fetus of unknown genotype. To note, in the case of an inherited X-linked disorder, cffDNA Sexing will be performed and RHDO analysis only carried out in the instance of a male pregnancy. (**B**) Informative single nucleotide polymorphisms (SNPs) spanning the region containing the variant of interest are ascertained by NGS and used to perform haplotype phasing of the low-risk (WT-associated) and high-risk (pathogenic variant-associated) alleles (several 100 kb in length). Informative SNPs are those where the parents are *not* heterozygous for the same genotype. For AR conditions, haplotype phasing is carried out using gDNA obtained from the mother, father and an affected proband. For X-linked conditions, phasing is performed using gDNA from the mother and an affected proband only. Note that for either mode of inheritance, a sample from an unaffected proband (i.e. homozygous WT) can be used instead if necessary (not depicted in the diagram). Unfilled and filled triangles represent informative SNPs associated with the unaffected or affected haplotype respectively. Unfilled and filled ovals depict the WT or pathogenic variant-of-interest, respectively. (**C**) RHDO is an indirect method for determining whether the fetus has inherited the low- or the high-risk allele, and is carried out on maternal plasma DNA. For AR conditions, the fetal genotype is determined based on 1) the relative abundance of SNPs associated with the maternal affected and unaffected haplotype, and 2) detection of SNPs associated with the paternal affected or unaffected haplotype using sequential statistical modelling, such as the SPRT. For X-linked conditions, the paternal haplotype is not required for RHDO analysis. (**D**) RMD is carried out as a direct method of measuring the relative abundance of the WT or pathogenic variant-of-interest in the cfDNA sample, using similar statistical tests as are required for RHDO.

RHDO involves linkage-based methods for variant calling whereby combined counts from thousands of heterozygous SNPs (otherwise referred to as informative SNPs) within the same haplotype surrounding the variant-of-interest are taken into consideration [8] (Figure 3). For autosomal recessive conditions, haplotype phasing of the risk-associated and WT alleles is carried out using NGS on genomic DNA obtained from the mother, father, and an affected or unaffected sibling (i.e. the proband) (Figure 3A,B). For X-linked conditions, the paternal sample is not required for phasing of the high- and low-risk alleles (Figure 3A,B). Sequencing analysis of the maternal plasma DNA is then performed, and SNPs are computationally assigned to a particular type according to their identity relative to the parental genotypes [8] (Figure 3C). Bayesian statistical modelling, using tests such as the sequential probability ratio test (SPRT), determines the relative increase or decrease in informative SNPs across the target haplotype, and thereby predicts fetal inheritance of the disease-associated, or WT, alleles [8] (Figure 3C). RHDO is currently clinically available for NIPD of the autosomal recessive disorders CF [39], SMA [41], and CAH [50], as well as X-linked *DMD*-related muscular dystrophies [40] (Figures 1 and 2). RHDO is still in the developmental setting for α- and β-thalassaemia (*HBA* and *HBB*) [51], retinoblastoma (*RB1*) [52], Ellis-van Creveld syndrome (*EVC*), factor 8 (*F8*) disorder/haemophilia A, and mucopolysaccharidosis type II (MPS II)/Hunter syndrome [53] (Figure 2).

A major advantage of RHDO is that it is not limited by the variant type. However, several challenges exist that have limited widespread implementation of this technology into the clinic. These include difficulties performing RHDO on consanguineous couples owing to the shared haplotype, and the requirement for a sample from both parents as well as an affected or unaffected proband or family member in order to perform the phasing analysis. Improvements to this approach are under investigation, including proband-free RHDO using long-read sequencing (Figure 1). Alternatively, RMD is a method which is highly sensitive and theoretically capable of diagnosing autosomal recessive and X-linked disorders requiring only a maternal blood sample [54] (Figures 1 and 2). Proband-free RHDO and RMD are discussed in more detail below.

## In the developmental pipeline

### Proband-free RHDO

Conventional RHDO relies on inferring the high-risk haplotype based on the SNP profile of an affected or unaffected proband; however, several direct haplotyping methods have recently been developed to facilitate parental haplotype phasing in circumstances where access to additional samples is unavailable. Two main approaches have been investigated for proband-free RHDO, including targeted locus amplification (TLA) [55,56] and microfluidics based linked-read sequencing (although the associated 10X Genomics technology is no longer available) [53,57–59]. As recommended by the American College of Obstetricians and Gynecologists (ACOG) [60], pre-pregnancy or prenatal carrier screening should be undertaken at a global population level for relatively common autosomal recessive disorders, such as SMA, CF and haemoglobinopathies. For these conditions, it is likely therefore that high-risk pregnancies will be identified in couples with no previous children, and therefore the development of universal direct-haplotyping tests could open up the possibility of RHDO for these first-time parents.

Recent advances in single molecule long-read sequencing platforms, such as those developed by Oxford Nanopore Technologies (ONT, Oxford, U.K.) [61] and Pacific Biosciences (PacBio, Menlo Park, CA, U.S.A.) [62], also have the potential to enable proband-free RHDO, as demonstrated in a recent study with relevance to β-thalassaemia, whereby ONT long-read sequencing was used to increase the length of individual haplotype blocks used for phasing analysis, and consequently improve statistical robustness [63]. In this study, Jiang and colleagues reported a failure to determine the maternal haplotype in one family as the closest heterozygous SNP was located outside of the PCR fragment boundary and thus not linkable to the variant-of-interest [63]. Further investigation is required to determine how common such local regions of homozygosity may be, and how this could negatively impact the utility of maternal haplotype phasing by long-read sequencing. PCR-related errors, including template-switching, should also be carefully considered with this approach.

cffDNA long-read sequencing has the potential to further increase the resolution and accuracy of fetal genotype classification as recombination events near the mutation site are more likely to be identified. Furthermore, the newly developed ONT readfish toolkit allows selective enrichment of reads-of-interest in real-time, which could assist with increasing sequencing depth specifically within the desired target regions [64]. Unfortunately, the error-rate of ONT is currently relatively high (∼5%) [65], although newer base calling models and chemistries are constantly being developed to improve accuracy towards levels that may be acceptable for clinical implementation. At present, PacBio sequencing offers greater accuracy, but the overall cost of this technology is higher than that of ONT which may preclude its use in publicly funded settings.

### RMD

RMD is an alternative method to RHDO, which has been investigated for the detection of maternally inherited variants in cfDNA. RMD is designed to directly detect single mutations, and operates under the assumption that there is a 50:50 ratio of the WT to pathogenic alleles in non-pregnant carriers [54] (Figure 3D). Similarly, if the mother and fetus share the same genotype, allelic balance is expected (Figure 3D). If the fetus is homozygous for the maternal pathogenic variant, however, a slight increase in the risk-associated allele relative to the FF will result, with the converse being true for a homozygous WT fetus (Figure 3D). For RMD to reliably replace invasive testing, the chosen method should be accurate at similar timepoints despite the low abundance of cffDNA in maternal circulation (i.e. at ∼11 weeks of gestation).

dPCR is the leading technology that has been applied to molecular counting [66–68], and its application in NIPT and NIPD research and development has been reviewed comprehensively elsewhere [69,70]. dPCR involves sample partitioning and target amplification within discrete reaction units thereby improving detection of rare variants within a high background of maternal plasma DNA, relative to conventional PCR-based methods, without the need for a standard curve [68,71,72]. To date, dPCR-based RMD for NIPD has not reached the clinic. However, this approach has shown clinical promise in several proof-of-concept studies for the detection of both paternally and maternally inherited variants, including for haemophilia [73,74], sickle cell disease [54,75], monogenic diabetes [76], β-thalassaemia [77–79], CF [80,81], inherited deafness [82], achondroplasia [83], and others [84,85] (Figure 2). The utility of haplotype-free RMD using dPCR is being considered for copy number variation (CNV) detection in autosomal recessive diseases, such as SMA, where gene copy number is negatively correlated with disease severity [86].

Previous generations of dPCR technologies were based on microfluidic devices with limited numbers of sample partitions [66,67]; however, several improvements have since resulted in the emergence of droplet dPCR (ddPCR) [87] (Figure 1). ddPCR is a highly sensitive, quantitative and high-throughput approach that involves separation of individual genomic targets into thousands (>10,000) of nanolitre-sized droplets using water–oil emulsion technology. The presence or absence of a variant-of-interest (i.e. WT vs pathogenic or maternal vs paternal) is ascertained on a droplet-by-droplet basis using fluorescence indicators.

RMD is clinically useful for detecting common pathogenic SNPs in the population using predesigned assays rapidly and at a low cost. A minimal FF threshold of >4% has typically been applied in the research setting using different methodologies for RMD-based testing for SNPs [74,86]. A major drawback of this approach that has delayed clinical implementation to date has been several reports of small numbers of incorrect and/or inconclusive results when using this technique [75–79,85]. For instance, Barrett and colleagues reported 5 misclassified (i.e. 1 false-positive and 4 false-negative) and 2 inconclusive results from a large cohort study of 59 individuals using ddPCR-based RMD for NIPD of sickle cell disease [75]. Sawakwongpra and colleagues similarly reported a misclassification rate of ∼20% (i.e. 5/24 cases) using this approach [78]. D’Aversa and colleagues reported 1 misclassified result out of 52 samples using ddPCR in the context of β-thalassaemia [77]. Perlado and colleagues also reported 1 false-positive and 5 inconclusive results [85]. Efforts to optimise the ddPCR methodology have been made by Constantinou and colleagues in their recent study which focused on RMD for β-thalassaemia; however, 1 misclassified and 6 inconclusive results out of 40 cases were still observed [79]. Insufficient FF (<4%) is believed to be a major contributor towards such discrepancies, although this cannot fully explain the results which have been described [83]. The minimal FF requirement for RMD using ddPCR is thus still somewhat uncertain. In addition to the FF, the total starting number of DNA molecules as well as the depth of the assay are also important contributors towards accurate genotype prediction using RMD. Purely setting a FF threshold is not necessarily a solution towards preventing incorrect and/or inconclusive calls. Such incorrect and inconclusive predictions, with as-of-yet inexplicable causes highlight the need for caution and further optimisation of the technology before NIPD using ddPCR-based RMD can be clinically implemented. Large-scale studies and machine learning approaches could improve the predictive value of RMD using dPCR in the future.

RMD has several advantages over RHDO, including that there is no requirement for paternal or proband samples, and that it is possible to detect *de novo* mutations provided the variant is suspected. Equally, however, RMD using dPCR has a number of disadvantages compared to RHDO. For instance, as RMD is focused only on a single mutation site while RHDO is haplotype-specific and covers lengthy genomic regions containing multiple SNPs, the possibility for multiplexing in a single assay is limited, and statistical robustness reduced. Furthermore, RMD is not applicable to large structural variants and mutations within highly repetitive regions or loci with similar sequence context elsewhere in the genome. As such, more sensitive approaches to RMD that are still achievable using only a maternal plasma sample are being investigated. These include circulating single-molecule amplification and resequencing technology (cSMART), which involves the introduction of UMI tags, circularization of tagged genomic DNA, inverse PCR, and sequencing. cSMART was developed as a proof-of-concept study for Wilson disease (*ATP7B*) using Sanger sequencing [88] and, more recently, has employed NGS technologies, showing clinical utility for autosomal recessive non-syndromic hearing loss (*GJB2* and *SLC26A4*) [89,90], β-thalassaemia (*HBB*) [91,92], methylmalonic acidaemia cblC type (*MMACHC*) [93], and phenylketonuria (*PAH*) [94]. Other studies have also described the use of barcode-enabled NGS to facilitate molecular counting with single base pair resolution [95–97], nested PCR and NGS [98], as well as size-based enrichment of cffDNA prior to diagnostic analysis [54,97].

### Fragmentomics and epigenetics

The term ‘fragmentomics’ has been adopted to describe the fragmentation properties of cfDNA, including the size distribution and biochemical nature of the free ends [99]. These assets can be leveraged to facilitate targeted analysis as well as enrichment of cffDNA. Additionally, the presence of longer cfDNA fragments provides a means to obtaining haplotype information from single cfDNA fragments using long-read sequencing technologies (PacBio and ONT) [13]. Long-read sequencing of cffDNA could therefore improve visualization of larger genomic aberrations, such as insertions, deletions and other sub-chromosomal structural rearrangements, and some triplet repeat expansions, which are difficult to detect by short-read sequencing. In addition, these longer fragments have enabled genetic-epigenetic tissue mapping on a single molecule level [9,13]. The abundance of CpG sites on the long cfDNA molecules means that single-molecule methylation analysis can be used to deduce the tissue of origin of individual plasma molecules. By first identifying cfDNA fragments containing the maternal-specific allele followed by methylation analysis to ascertain whether or not this allele is present on placenta-derived cfDNA molecules, the maternal inheritance of the fetus can be determined without the need for the sensitive molecular counting and dosage-based techniques previously described for both RHDO and RMD. The proof-of-principle study demonstrating this technique has been carried out using SMRT sequencing [13], which currently carries a high cost. However, as sequencing costs continue to decline and with the improving accuracy of ONT sequencing this approach will no doubt become viable for clinical practice in the future.

### Cell-based approaches

Fetal DNA in maternal circulation does not only exist in the cell-free, fragmented form, as fetal and placental cells are trafficked into the maternal bloodstream from as early as 4–6 weeks of pregnancy and have been successfully isolated during these early timepoints [100,101]. An advantage of a cell-based source of fetal DNA is that the genomic material remains intact, thereby circumventing some of the challenges experienced with short-read cfDNA such as the limited size of variants that can be detected and difficulties with haplotype phasing [102] (Figure 1). Four major populations of fetal cells that are trafficked through the placenta into the bloodstream have been detected. Trophoblasts and nucleated red blood cells (early erythrocytes) (reviewed in [103]) exist transiently in circulation, are thus specific to the ongoing pregnancy, and provide a pure population of fetal or placental cells (fetal cells holding an advantage since they are not subject to confined placental mosaicism). The two other major classes of circulating cells are fetal lymphocytes and stem/progenitor cells, however these were found to persist for several years following birth and so have garnered less attention for cell-based NIPD [104].

One of the major limitations of a cell-based approach that has hindered its clinical implementation to date is rooted in the relatively low abundance of circulating fetal cells within the high background of nucleated maternal haematopoietic cells [102,105]. For instance, a recent study reported isolation of, on average, 2.5 cells per mL of blood across 8 cases [106]. Unlike the FF of cfDNA which increases with pregnancy progression, the optimal window for collection of fetal cells from the maternal bloodstream is believed to be ∼10–14 weeks, during which time there is a high level of placental vascularisation [103]. Techniques are therefore required for the enrichment of fetal/trophoblastic cells, which can also be done alongside depletion of maternal cells. After enrichment, isolation of fetal cells then requires high-throughput processing for the detection of multiple fetal cell markers. Recently, a combined effort between medical genetics and materials science has enabled innovative advancements towards improving detection, enrichment, isolation and characterization of cell-based fetal DNA [107].

Another limitation of these cell-based approaches is that whole genome amplification is required prior to single cell sequencing, and this makes this technique susceptible to significant allele dropout and amplification errors [108]. It is feasible that optimization of isolation methods may overcome the challenge of low yields and a relatively narrow sampling window; however, the techniques are both time-intensive and costly for clinical implementation, and currently none of the many published methods of cell selection and identification have been validated for clinical use [103]. Trophoblast retrieval and isolation from the cervix (TRIC) is an alternative, semi-invasive method to obtain fetal cells which has been proposed to improve yields without a known associated risk of miscarriage [109,110]. Extraction of mononuclear extravillous trophoblasts (EVTs) by TRIC was reported to provide ∼500–1500 cells per sample, far greater than from maternal peripheral blood samples. A possible limitation towards the clinical implementation of TRIC is that trained personnel are required for cell collection from the cervix, although this is not too dissimilar to commonly performed cervical cancer screening methods.

While cell-based NIPD has great promise in clinical practice to be a useful alternative to CVS and amniocentesis, much work is still required to improve the methods for high-throughput isolation of pure fetal cell populations and to ensure consistent test performance at low cost.

## Commercial screening for low-risk pregnancies

NIPD is currently commercially available for low-risk pregnancies (Figures 1 and 2). One such screening test, marketed under the brand name Vistara (Natera, Inc., San Carlos, CA, U.S.A.), screens for 25 autosomal dominant and X-linked conditions across 30 genes using a unique molecular indexing (UMI) technique for the NGS analysis [111] (Figure 2). This test requires genomic DNA from both parents to be tested alongside the cfDNA in order to assist with the interpretation, and it cannot detect variants which are maternally inherited (including those which are also X-linked). A recent review of this service looked at the results for 2208 women, of which 125 (5.7%) were positive [112]. The authors report no false-positive or -negative results, with a follow-up rate of 53.6%. It is important to take note of this low rate of follow-up in this study, reflected in a statement made by ACOG regarding single gene cfDNA screening, which states that there is insufficient data to provide information regarding the positive and negative predictive value in the general population, and therefore it does not currently recommend this screening in pregnancy [60]. In addition to the lack of long-term follow-up, it should also be noted that the minimum NGS coverage requirements for the target regions is ≥97% [111,112]. Although this information is provided in the literature, the majority of patients will likely be unaware that coverage of certain genes may not be 100%, and the impact that this could have on the interpretation of their result. Of note, the minimal FF requirement for this test is 4.5% [112].

Another commercially available screening test is the UNITY Screen™ (BillionToOne Inc., Menlo Park, CA, U.S.A.) (Figure 2). This test offers a maternal carrier screen for four recessive conditions, including CF, SMA, and haemoglobinopathies, as recommended by ACOG [60], with reflex cfDNA testing for all those identified as carriers [95]. This RMD-based screening test, which uses a Quantitative Counting Template (QCT) molecular counting approach rather than UMIs, can therefore provide a personalised fetal risk assessment without the need for a paternal sample. While the minimum FF required for the UNITY Screen™ is not explicitly stated on the company website, reliable results were reported to be obtained at a FF ≥ 5%, according to their recent publication [95]. With respect to CF and the group of inherited haemoglobinopathies, the UNITY Screen™ is not fully exhaustive for all disease-causing recessive variants within the *CFTR* and *HBA*/*HBB* genes, respectively (Figure 2). Even so, a recent review of this carrier screen with reflex single gene NIPT identified that, as first-line screening, it could offer significant cost savings for the healthcare system when compared to traditional carrier screening, and also a 2.4-fold improvement in the detection of affected fetuses [113]. The authors go on to state, however, that additional studies will be required to further assess the clinical utility, and there is as yet no support from national bodies for such screening based on analysis of cfDNA to be implemented.

Commercial availability of NIPD tests offers expansion of the service to include low-risk pregnancies; however, a growing concern in the health-care community is that expert pre- and post-test counselling may not be available to patients. Interpretation of results is essential to take into consideration the possibility of factors such as variable disease expressivity, incomplete penetrance, incomplete gene coverage, and risks of false-negative or false-positive results. Furthermore, this should be clearly distinguished from NIPD offered in pregnancies at known increased risk because of a family history or ultrasound findings. In the latter situation, cfDNA testing is diagnostic and does not require confirmation of positive findings by analysis of a fetal sample obtained by invasive testing. If the commercial test is offered in low-risk pregnancies, invasive testing will be required to confirm high-risk results, at least until sufficient data are available to demonstrate the sensitivity, specificity and positive predictive value of these tests.

## Conclusions

The ability to examine the fetal genotype at single nucleotide resolution from within maternal plasma DNA has revolutionised prenatal care. A plethora of approaches have been developed for low-level variant detection for paternally inherited and *de novo* variants, and dosage-based and molecular counting techniques for the detection of maternal variants. Proof-of-concept studies indicate that several additional methods for NIPD of monogenic disease have clinical utility, notably the discovery of long cfDNA fragments, and the next phase will be critical for bridging the gap between the research and clinical settings. While no single strategy will cover all possible rare diseases, keeping apace with technological advancements, streamlining workflows and reducing the overall cost of testing will be essential for the expansion of NIPD for monogenic disease in the clinic, but this can be challenging given the rarity of many conditions. Importantly, the minimal FF, starting genomic material, and depth of assay required across the various NIPD methodologies in order to obtain consistently accurate fetal genotype predictions across all modes of inheritance require deeper investigation.

Even if the possibility exists to carry out NIPD earlier in pregnancy than for invasive testing, there needs to be confidence that consistently high levels of sensitivity and specificity will be obtained. This could vary depending on the suspected diagnosis, and the chosen method for carrying out NIPD. There is an ongoing debate as to who should have access to testing, and the burden of information on patients as well as clinical researchers must be carefully considered prior to the implementation of new tests into the clinic. In addition, even with clear selection guidelines, disparities with regards to access and availability of resources must be taken into account so as to reduce inequalities. Conversations across multidisciplinary teams is essential for relevant case selection and to balance clinical resources with diagnostic yield. With the rapid strides being made in gene therapy for rare monogenic disorders, the panel of genes and diseases we are able to include is ever-growing, if we are to work towards accurate diagnosis and treatment of affected children from the earliest timepoint.

## Data Availability

There is no data available for sharing relating to this manuscript.

## Open Access

Open access for this article was enabled by the participation of University College London in an all-inclusive *Read & Publish* agreement with Portland Press and the Biochemical Society under a transformative agreement with JISC.

## Abbreviations

ACOG: American College of Obstetricians and Gynecologists
AD: autosomal dominant
AR: autosomal recessive
BMD: Becker muscular dystrophy
CAH: congenital adrenal hyperplasia
CF: cystic fibrosis
cfDNA: cell-free DNA
cffDNA: cell-free fetal DNA
CNV: copy number variation
CPM: confined placental mosaicism
cSMART: circulating single-molecule amplification and resequencing technology
CVS: chorionic villus sampling
ddPCR: droplet dPCR
DMD: Duchenne muscular dystrophy
dPCR: digital polymerase chain reaction
FF: fetal fraction
NGS: next generation sequencing
NIPD: non-invasive prenatal diagnosis
NIPT: non-invasive prenatal testing
ONT: Oxford Nanopore Technologies
PacBio: Pacific Biosciences
PCR-RED: PCR-restriction enzyme digestion
qPCR: quantitative polymerase chain reaction
RMD: relative mutation dosage
RHD: rhesus D blood group
RHDO: relative haplotype dosage analysis
SMA: spinal muscular atrophy
SNP: single nucleotide polymorphism
SPRT: sequential probability ratio test
TLA: targeted locus amplification
TRIC: trophoblast retrieval and isolation from the cervix
WT: wildtype
